# Supplementary material for: Willingness to adopt green house gas mitigation measures: Agricultural land managers in the United Kingdom
Source: PLoS One. 2024 Jul 8;19(7):e0306443. doi: 10.1371/journal.pone.0306443 (PMC11230571; doi:10.1371/journal.pone.0306443)
Supplement: S1 Fig — (DOCX) [file pone.0306443.s001.docx]

S1 Figure. Figure of the frequency distribution of willingness to try new measures for reducing GHG emissions.
