# Supplementary material for: Willingness to adopt green house gas mitigation measures: Agricultural land managers in the United Kingdom
Source: PLoS One. 2024 Jul 8;19(7):e0306443. doi: 10.1371/journal.pone.0306443 (PMC11230571; doi:10.1371/journal.pone.0306443)
Supplement: S1 Table — (DOCX) [file pone.0306443.s002.docx]

**S1 Table.** Frequency Distribution of Agricultural Land Managers’ Socioeconomic Data and GHGs Emission Measures

Table 1. Frequency Distribution of Agricultural Land Managers’ Age

| Age | Number | % |
| --- | --- | --- |
| 18-24 | 5 | 2.5 |
| 25-39 | 66 | 33.5 |
| 40-49 | 72 | 36.5 |
| 50-59 | 39 | 19.8 |
| 51-60 | 0 | 0.0 |
| 61-70 | 11 | 5.6 |
| 71+ | 4 | 2.0 |
|  | 197 | 100 |

Table 2. Frequency Distribution of Number of Full-time Equivalent (FTEs) Workers on the farm

| Number of FTEs | Count | Percentage |
| --- | --- | --- |
| less than 0.5 | 8 | 4.1 |
| 0.5-1 | 22 | 11.2 |
| 1-1.9 | 44 | 22.3 |
| 2-2.9 | 45 | 22.8 |
| 3-4.9 | 27 | 13.7 |
| 5+ | 51 | 25.9 |
|  | 197 | 100 |

Table 3. Distribution of Dominant farm enterprise (on the average represents >2/3rds of farmers output)

| Farming sector | Distribution of respondents | Percentage |
| --- | --- | --- |
| Other | 2 | 1 |
| Horticulture | 10 | 5 |
| Specialist Poultry | 14 | 7 |
| Dairy | 13 | 6.5 |
| Lowland Grazing Livestock | 14 | 7 |
| Specialist Pigs | 14 | 7 |
| Grazing Livestock (Less favoured areas) | 15 | 7.5 |
| General cropping | 29 | 15 |
| Mixed | 31 | 16 |
| Cereals | 54 | 27 |

Table 4. Agricultural Land Managers’ Time in Farming

| Time in farming | Count | % |
| --- | --- | --- |
| 0-5 years | 10 | 5.1 |
| 6-10 years | 45 | 22.8 |
| 11-20 years | 41 | 20.8 |
| 21-30 years | 51 | 25.9 |
| 31-40 years | 21 | 10.7 |
| 41-50 years | 16 | 8.1 |
| 51+ years | 13 | 6.6 |

Table 5. Distribution of Agricultural Land Managers’ Highest Level of Education

| Level of education | Count | % |
| --- | --- | --- |
| below GCSE / O-Level | 7 | 3.6 |
| GCSE / O-Level | 12 | 6.1 |
| HND / Other diploma | 25 | 12.7 |
| A-Level | 27 | 13.7 |
| Undergraduate degree | 83 | 42.1 |
| Formal apprenticeship | 22 | 11.2 |
| Postgraduate degree | 18 | 9.1 |
| Other | 3 | 1.5 |

Table 6. Distribution of perception of current business performance as an income-generating business.

| Current business performance | Count | % |
| --- | --- | --- |
| Very well | 44 | 22.6 |
| Fairly well | 69 | 35.4 |
| Average | 65 | 33.3 |
| Not very well | 15 | 7.7 |
| Very poorly | 2 | 1.0 |

Table 7. Distribution of Motivation to take up environmental practices

| Motivations | Count | Percentage |
| --- | --- | --- |
| Opportunity to reduce GHG emissions | 72 | 14.9 |
| Improving soil health | 91 | 18.8 |
| The opportunity to leave a legacy | 57 | 11.8 |
| Increasing efficiency | 36 | 7.4 |
| To boost biodiversity | 72 | 14.9 |
| Increasing profitability | 60 | 12.4 |
| Adapting for the future | 23 | 4.8 |
| Improving water quality | 41 | 8.5 |
| Receiving funding from an agri-environment scheme | 32 | 6.6 |

Table 8. Distribution of Willingness to try new measures for reducing GHG emissions

| Willingness to try new measures for reducing GHG emissions | Count | Percentage |
| --- | --- | --- |
| Very willing | 68 | 35.1 |
| Quite willing | 79 | 40.7 |
| Neutral | 35 | 18.0 |
| Quite unwilling | 7 | 3.6 |
| Very unwilling | 5 | 2.6 |
