# Supplementary material for: Willingness to adopt green house gas mitigation measures: Agricultural land managers in the United Kingdom
Source: PLoS One. 2024 Jul 8;19(7):e0306443. doi: 10.1371/journal.pone.0306443 (PMC11230571; doi:10.1371/journal.pone.0306443)
Supplement: S2 Table — (DOCX) [file pone.0306443.s003.docx]

**S3 Table. Test for Stepwise regression predictors selection**

Table 1. Test for Stepwise regression predictors selection with Mean Willingness to Adopt as the outcome variable

| **Number of Predictors** | **Predictors for each model (Mean Willingness to Adopt)** | **R2ADJ** | **C** | **AIC** | **AICC** | **BIC** |
| --- | --- | --- | --- | --- | --- | --- |
| 1 | dom_grazinglivestock | 0.224 | 55.448 | 204.778 | 204.915 | 211.153 |
| 2 | workfte dom_grazinglivestock | 0.282 | 39.239 | 192.044 | 192.274 | 201.606 |
| 3 | workfte anaerobic dom_grazinglivestock | 0.328 | 26.375 | 181.048 | 181.394 | 193.797 |
| 4 | workfte dom_farmcereal anaerobic dom_grazinglivestock | 0.359 | 18.147 | 173.523 | 174.011 | 189.460 |
| 5 | workfte dom_farmcereal gen_cropdom anaerobic dom_grazinglivestock | 0.397 | 8.230 | 163.745 | 164.400 | 182.869 |
| 6 | workfte dom_farmcereal gen_cropdom anaerobic dom_grazinglivestock age_below50 | 0.415 | 3.963 | 159.227 | 160.074 | 181.538 |
| 7 | workfte dom_farmcereal gen_cropdom anaerobic dom_grazinglivestock educ_bi age_below50 | 0.424 | 2.301 | 157.304 | **158.369** | 182.803 |
| 8 | workfte dom_farmcereal gen_cropdom anaerobic dom_grazinglivestock educ_bi age_below50 farmpercepwell | **0.428** | 2.309 | **157.134** | 158.443 | 185.820 |
| 9 | workfte dom_farmcereal gen_cropdom anaerobic dom_grazinglivestock educ_bi age_below50 farmpercepwell rentlandha | 0.427 | 3.602 | 158.357 | 159.938 | 190.231 |
| 10 | workfte dom_farmcereal gen_cropdom anaerobic dom_grazinglivestock educ_bi age_below50 farmpercepwell rentlandha dom_mixedfarm | 0.426 | 4.986 | 159.677 | 161.557 | 194.739 |
| 11 | workfte dom_farmcereal gen_cropdom anaerobic dom_grazinglivestock educ_bi age_below50 farmexp21_40yrs farmpercepwell farmexp0_20yrs rentlandha | 0.425 | 6.150 | 160.751 | 162.957 | 199.000 |
| 12 | workfte dom_farmcereal gen_cropdom anaerobic dom_grazinglivestock educ_bi age_below50 farmexp21_40yrs farmpercepwell farmexp0_20yrs rentlandha dom_mixedfarm | 0.423 | 7.673 | 162.221 | 164.782 | 203.657 |
| 13 | workfte dom_farmcereal gen_cropdom anaerobic dom_grazinglivestock educ_bi age_below50 farmexp21_40yrs farmpercepwell farmexp0_20yrs rentlandha dom_mixedfarm ownershipland | 0.421 | 9.349 | 163.859 | 166.804 | 208.483 |
| 14 | workfte dom_farmcereal gen_cropdom anaerobic dom_grazinglivestock educ_bi age_below50 farmexp21_40yrs farmpercepwell farmexp0_20yrs rentlandha dom_mixedfarm ownershipland ownlive > stock | 0.418 | 11.080 | 165.559 | 168.917 | 213.370 |
| 15 | workfte dom_farmcereal gen_cropdom anaerobic dom_grazinglivestock educ_bi age_below50 farmexp21_40yrs farmpercepwell farmexp0_20yrs rentlandha dom_mixedfarm ownershipland ownlive > stock ownpoultry | 0.415 | 13.030 | 167.504 | 171.305 | 218.502 |
| 16 | workfte dom_farmcereal gen_cropdom anaerobic dom_grazinglivestock educ_bi age_below50 farmexp21_40yrs farmpercepwell farmexp0_20yrs rentlandha dom_mixedfarm ownershipland ownlive > stock ownpoultry farmedareaha | 0.411 | 15.020 | 169.492 | 173.767 | 223.677 |
| 17 | workfte dom_farmcereal gen_cropdom anaerobic dom_grazinglivestock educ_bi age_below50 farmexp21_40yrs farmpercepwell farmexp0_20yrs rentlandha dom_mixedfarm ownershipland ownlive  > stock ownpoultry grow_covercrop farmedareaha | 0.408 | 17.007 | 171.478 | 176.257 | 228.850 |
| 18 | workfte dom_farmcereal gen_cropdom anaerobic dom_grazinglivestock educ_bi age_below50 farmexp21_40yrs farmpercepwell farmexp0_20yrs rentlandha dom_mixedfarm ownershipland ownlive  > stock ownpoultry grow_covercrop farmedareaha far_percepaverage | 0.404 | 19.000 | 173.470 | 178.786 | 234.030 |

Table 2. - Test for Stepwise regression predictors selection with PCA scores as the outcome variable

| **Number of predictors** | **Predictors for each model (PCA Scores )** | **R2ADJ** | **C** | **AIC** | **AICC** | **BIC** |
| --- | --- | --- | --- | --- | --- | --- |
| 1 | dom_grazinglivestock | 0.202 | 38.372 | 526.189 | 526.397 | 531.747 |
| 2 | workfte dom_grazinglivestock | 0.290 | 22.284 | 513.255 | 513.606 | 521.593 |
| 3 | workfte anaerobic dom_grazinglivestock | 0.346 | 12.589 | 504.496 | 505.026 | 515.612 |
| 4 | workfte dom_farmcereal anaerobic dom_grazinglivestock | 0.385 | 6.204 | 498.135 | 498.885 | 512.030 |
| 5 | workfte dom_farmcereal gen_cropdom anaerobic dom_grazinglivestock | 0.420 | 0.683 | 492.101 | 493.110 | 508.775 |
| 6 | workfte dom_farmcereal gen_cropdom anaerobic dom_grazinglivestock age_below50 | 0.439 | -1.847 | **488.986** | **490.295** | **508.440** |
| 7 | workfte dom_farmcereal gen_cropdom anaerobic dom_grazinglivestock educ_bi age_below50 | **0.441** | -1.060 | 489.579 | 491.231 | 511.812 |
| 8 | workfte dom_farmcereal gen_cropdom anaerobic dom_grazinglivestock educ_bi age_below50 farmpercepwell | 0.441 | 0.059 | 490.546 | 492.583 | 515.558 |
| 9 | workfte dom_farmcereal gen_cropdom anaerobic dom_grazinglivestock educ_bi age_below50 farmpercepwell rentlandha | 0.438 | 1.694 | 492.116 | 494.583 | 519.907 |
| 10 | workfte dom_farmcereal gen_cropdom anaerobic dom_grazinglivestock educ_bi age_below50 farmpercepwell rentlandha dom_mixedfarm | 0.433 | 3.518 | 493.907 | 496.850 | 524.477 |
| 11 | workfte dom_farmcereal gen_cropdom anaerobic dom_grazinglivestock educ_bi age_below50 farmexp21_40yrs farmpercepwell farmexp0_20yrs rentlandha | 0.429 | 5.290 | 495.637 | 499.104 | 528.987 |
| 12 | workfte dom_farmcereal gen_cropdom anaerobic dom_grazinglivestock educ_bi age_below50 farmexp21_40yrs farmpercepwell farmexp0_20yrs rentlandha dom_mixedfarm | 0.425 | 7.143 | 497.463 | 501.501 | 533.591 |
| 13 | workfte dom_farmcereal gen_cropdom anaerobic dom_grazinglivestock educ_bi age_below50 farmexp21_40yrs farmpercepwell farmexp0_20yrs rentlandha dom_mixedfarm ownershipland | 0.420 | 9.087 | 499.396 | 504.056 | 538.303 |
| 14 | workfte dom_farmcereal gen_cropdom anaerobic dom_grazinglivestock educ_bi age_below50 farmexp21_40yrs farmpercepwell farmexp0_20yrs rentlandha dom_mixedfarm ownershipland ownlive > stock | 0.414 | 11.051 | 501.353 | 506.686 | 543.040 |
| 15 | workfte dom_farmcereal gen_cropdom anaerobic dom_grazinglivestock educ_bi age_below50 farmexp21_40yrs farmpercepwell farmexp0_20yrs rentlandha dom_mixedfarm ownershipland ownlive > stock ownpoultry | 0.409 | 13.010 | 503.304 | 509.364 | 547.770 |
| 16 | workfte dom_farmcereal gen_cropdom anaerobic dom_grazinglivestock educ_bi age_below50 farmexp21_40yrs farmpercepwell farmexp0_20yrs rentlandha dom_mixedfarm ownershipland ownlive > stock ownpoultry farmedareaha | 0.403 | 15.004 | 505.298 | 512.138 | 552.543 |
| 17 | workfte dom_farmcereal gen_cropdom anaerobic dom_grazinglivestock educ_bi age_below50 farmexp21_40yrs farmpercepwell farmexp0_20yrs rentlandha dom_mixedfarm ownershipland ownlive > stock ownpoultry grow_covercrop farmedareaha | 0.397 | 17.004 | 507.298 | 514.975 | 557.322 |
| 18 | workfte dom_farmcereal gen_cropdom anaerobic dom_grazinglivestock educ_bi age_below50 farmexp21_40yrs farmpercepwell farmexp0_20yrs rentlandha dom_mixedfarm ownershipland ownlive > stock ownpoultry grow_covercrop farmedareaha far_percepaverage | 0.391 | 19.000 | 509.293 | 517.864 | 562.096 |
