# Supplementary material for: Willingness to adopt green house gas mitigation measures: Agricultural land managers in the United Kingdom
Source: PLoS One. 2024 Jul 8;19(7):e0306443. doi: 10.1371/journal.pone.0306443 (PMC11230571; doi:10.1371/journal.pone.0306443)
Supplement: S3 Table — (DOCX) [file pone.0306443.s004.docx]

S4 Table. Quotes and paraphrasing of farmers’ explanation of willingness to adopt GHG mitigation measures.

| **Table 1. Quotes and paraphrasing of farmers’ explanation for willingness to decrease use of nitrogen fertilisers** |
| --- |
| - The effect seems well worth trying |
| - It's something we are already doing |
| - Protecting the environment is the responsibility of citizens, Environmental protection |
| - currently using very little due to cost, we already have reduced use |
| - Improve efficiency while being environmentally friendly |
| - I have already reduced my N fertiliser use by 2/3rds |
| - If my produce will be worth 3 times as much of my yields halve, then I’m interested, but who’s paying the difference? I’m very sceptical that this is going to do anything other than make our business less viable. |
| - Clean air |
| - I keep all inputs to a minimum. I find that by keeping soil in good condition, longest possible grazing intervals and especially keeping pH optimum with local sea sand gives me enough forage. |
| - Wiling to try as long as it is proved not to harm our profitability. |
| - I would love to cut costs. It'll save me money |
| - Environmental protection |
| - Have only just dropped out of being organic due to poor profit margins available |
| - I am organic, and almost completely pasture fed (rotational grazing), so I think I am probably almost if not already net zero in my farming; I stopped using nitrogen fertiliser about 7yrs ago, I’m organic using clover fiction instead |
| - Nitrogen fertiliser is our biggest contribution to GHG emissions and water pollution. I also believe that, if we could find alternative forms of crop nutrition, it could improve our crop health and reduce incidence of pests and diseases and our reliance on pesticides |
| - Depends on the price of Nitrogen fertiliser and decision made on profitability. |
| - Would like to grow more legumes (no nitrogen needed) and more crop rotation but cost of fencing and previous generation hinder. |
| - Realistically reducing N is the single biggest factor in reducing the GHG emissions of almost any conventional arable farm - and doing so will help reduce one of our biggest costs. |
| - We already have reduced N fertiliser use. We built a large slurry lagoon so that we can store more slurry and then make better use of it, thereby reducing the need for N fertiliser. This was a good move ahead of the increase in fertiliser prices! We are not large users of inorganic N at all, but we're always looking to only use what we have to. |
| - We’ve already had to reduce due to price of fertiliser. It hasn’t been great for us this summer although some of that may be the drought. Not having enough Fert means we may not have enough food for our stock and could have to buy in from someone who does have enough Fert. I’m not sure that’s a great thing to do. Exploring how much we could reduce without adverse effect would be interesting. |
| - As a beef and sheep farmer fertiliser has become too expensive to apply at previous rates. So it’s use has been cut back drastically |

| **Table 2. Quotes and paraphrasing of farmers’ explanation for willingness to reduce livestock stocking density and pesticides use** |
| --- |
| - Already reduced cow numbers, mob graze, use herbal leys, much reduced N on other grasses, extended grazing season,   I am reducing stocking density as more extreme weather is shortening my grazing season for various reasons. I am experimenting with agroforestry (nut trees) as I think it is important to produce nutrient dense foods in a sustainable way. |
| - Love environmental protection, I am willing to try to improve the environment. |
| - I'm getting the feeling that the person setting the questions doesn't understand the difference between warming impact and the arbitrary concept of "emissions". You seem to be under the illusion that emissions = warming impact. This is NOT the case. I'll assume you don't actually know what I'm on about here and see if I'm proved correct as we go. |
| - Pointless exercise, as GHG are not being caused by livestock... |
| - Don’t use pesticide and i use grass in the most efficient way. If i have less livestock i would not be efficient. |
| - Reducing organic stocking rates would have no effect on decreasing carbon, in fact, it would reduce carbon sequestration into my soil |
| - Having moved to a 'low input' system I've already reduced the overall livestock numbers to account for this. |
| - I am the integration of food and livestock, they play a role in promoting each other, manure made of organic fertilizer into the field, crop straw to do their feed, livestock and poultry reduced, fertilizer is not enough, need one |
| - If I lose one, I reduce my income |
| - Poorer grassland performance may result from undergrazing |
| - Already have by putting arable land into herbal leys to improve soils |
| - Not sure if reducing numbers would be the best for grassland management |

| **Table 3. Quotes and paraphrasing of farmers’ explanation for willingness to use anaerobic digestion as a way of reducing GHG emissions.** |
| --- |
| - An AD plant wouldn’t be very economic due to small number of cattle |
| - to expensive for small scale farm, cost is a problem, It doesn't feel very cost-effective |
| - Environmental protection |
| - Not willing to |
| - We are predominantly a cereal/veg farm, so to move to anaerobic digestate means a considerable investment in infrastructure for which the returns are not there. I would also question the green credentials of AD, is the carbon footprint of the infrastructure manufacturing taken into account anywhere? It is a very energy intense operation growing and harvesting the crop and I have serious doubts whether energy produced is greater than total energy consumed. |
| - Not sure how burning diesel to produce a crop to make gas to be burnt in a engine will reduce GhG |
| - Not sure how I could use it. |
| - Opportunity to increase profitability and reduce carbon footprint sounds good. |
| - Love environmental protection, I am willing to try to improve the environment. |
| - At my scale I would be happy to experiment on a domestic scale version. It would need to be the 'dry' feedstock version. |
| - I'm probably not big enough or have enough slurry to make digestion viable in my own right. However, if capital grants were available to build a small scale digester I'd consider hosting the digester & using slurry from my neighbours. |
| - I can’t afford to install AD and doubt I’ve got enough adult cattle to service it. We also don’t have 3 phase electricity anywhere nearby, |

| **Table 4. Quotes and paraphrasing of farmers’ explanation for willingness to supplement feed to improve the efficiency of feed utilisation and reduce ammonia/enteric methane emissions in their urine.** |
| --- |
| - Ruminants are part of a natural cycle. Please look it up. As soon as you talk about additives, supplements etc you are engaging multinational supply chains, which are not a force for good. |
| - Would have to be suitable for adding to hay at the point of feeding during 5 months housing and pay for itself. Would like to have knowledge of the whole impact of producing/using supplement. |
| - I am willing to try to improve the environment. |
| - Being organic I feed only grass. No concentrates, so I can’t reduce it! |
| - Don't believe the figures. How can it be better to buy all these products, load them on a lorry, transport it who knows how far, unload it, store it, and then mix it rather than feeding our own home produced grass & grain/beans |
| - We only feed dry materials in winter, add some bean cake, rapeseed meal, etc., to supplement their energy, livestock and poultry can be healthy, other times are grazing, mountain grass also has licorice growth, licorice has the effect of antitoxin |
| - But not heard of it. |
| - One is in the dark about the costs. If I spend a £1, I need to get it back. |
| - I need to see a proven reduction for the investment made, and someone needs to pay for it if I do reduce otherwise why bother. I also need proof it is safe for my livestock |
| - Definitely willing to do this if there are benefits to feed conversion as well as reductions in GHG emissions |
| - Hopefully it will reduce the cost |

| **Table 5. Quotes and paraphrasing of farmers’ explanation for willingness increase the extent to which they monitor performance of livestock** |
| --- |
| - We already performance record from birth and select replacements on efficiency. But could always do more |
| - Increase Revenue |
| - I am willing to try to improve the environment. |
| - According to many years of breeding experience, in each livestock estrus season, we send more experienced people to participate in grazing, monitor the estrus date of the animals, record it, and decide the mating time |
| - More data on performance can help efficiency |
| - I’m struggling to keep up with the data input, it all takes a lot of time and there’s no one central point. By the time I’ve inputted the data there is never anytime to analyse it. |
| - If this can show an economic return happy to consider this |

| **Table 6. Quotes and paraphrasing of farmers’ explanation for willingness to apply additional soil amendments (basalt, biosolids) on cropped land** |
| --- |
| - Not willing |
| - Biosolids - micro plastics, pharmaceuticals, no thank you. I can see the headlines 10 yrs down the line “use of biosolid fertiliser linked to cancer, antibiotic resistance, declining birth rates…..” etc. And it’ll be spun as the greedy ignorant yokels playing fast and loose with public health. Again, same as the Pirbright foot and mouth leaks. I want to improve our soil by growing the solution, not buying it. If someone is saying the answer is a bagged product, which they happen to be selling, or have been paid to research, then I’m afraid I’m a sceptic. |
| - I don't have cropped land. I wouldn't take biosolids as have concerns about micro plastics. Although long term perm pasture a lot of my ground is disturbed by 19c mining and I think soil carbon is probably still building |
| - Returning OM to the soil is essential for regenerating SOM/SOC stocks depleted in the past 70 years. Also an important means of reducing reliance on synthetic fertilisers & building soil health & structure. |
| - If there is no direct cost I would be keen. How would it work? are these products delivered and spread free without any carbon foot print. |
| - I am worried about micro plastics from bio solids, I already apply trace elements where needed, but I think great improvements can be had just from improved grazing management anyway, that would be the low hanging fruit before buying anything in. |
| - Bio solids are expensive, application can be problematic, and they contain microplastics, not something I would apply to my land. Acquiring something like basalt which is not a local product would cost more in the mining, processing and transport than the benefit it would result in. |
| - Already applying biosolids. Would consider other amendments if there really is a benefit to soil quality. |

| **Table 7. Quotes and paraphrasing of farmers’ explanation for willingness to apply nitrification inhibitors to your soils** |
| --- |
| - Not willing, I don’t know the actual effect; |
| - unsure if this is needed on our soil type or our organic livestock system? |
| - An important means of reducing farm GHGs and ensuring nitrogen use efficiency/reducing pollution, but - they do come at a cost which is borne by the farmer. |
| - I am willing to try to improve the environment. |
| - Has to prove cost effective |
| - Because the fertiliser containing nitrate element, easy to produce explosion, dangerous, dare not use |
| - Don’t know anything about this. |
| - I doubt it is the best return on investment from a GHG or economic savings perspective |
| - Need more info |

| **Table 8. Quotes and paraphrasing of farmers’ explanation for willingness to introduce legumes to grasslands and/or crop rotation.** |
| --- |
| - Not willing |
| - At present my management is encouraging wild legumes. We did introduce red clover seeds on patches left by spot spraying thistles. They have spread and persisted on the long grazing/hay regime |
| - All the discussion around legumes seems to assume that they work in the same way as nitrogen fertiliser. They don't. They contribute a reasonable amount of nitrogen to companion plants but only if zero fertiliser is applied. They also take time to get going in the spring, which unfortunately doesn't coincide with the companion plants' spring demands. So, all of your figures are simply extrapolation of best-case scenarios which is why huge errors occur when policies are based on such calculations. That's not to say I am anti legumes as such. Escalation of fertiliser prices is going to produce some "interesting" results in the future. |
| - We are already planting mixed swards. |
| - It's a viable solution. Soybeans have rhizobia clumps, they fix nitrogen, they're good for other crops, and we're going to do that. We're going to interplant soybeans with corn and other tall crops |
| - They come with challenges. If they were truly economical alongside grass more people would be doing it. Early growth is a challenge. Weed control with loss of certain agrochemicals is also a challenge |
| - Already doing this and plan to do more |

| **Table 9. Quotes and paraphrasing of farmers’ explanation for willingness to introduce fresh grass your cattle consume?** |
| --- |
| - We are already a grass-based system using as much of our own grass as possible supplemented with home grown grain/beans. |
| - In addition to grazing weeds in the mountains, the fields that could not grow food were used to plant a variety of herbage, which was harvested, and fed. Dry material was fed only when the door was closed by rain and snow. |
| - Not willing. |
| - I am already rotation grazing, and am learning gradually to do it better. |
| - Grazing efficiency is the key to ruminant profitability. |
| - Yes, it can improve profitability. |

| **Table 10. Quotes and paraphrasing of farmers’ explanation for willingness to grow cover crops and switch to reduced/minimum tillage?** |
| --- |
| - Protect the land |
| - I have received a lot of soil run off from up slope neighbour’s arable land. He is donating quite a lot of his topsoil where it settles in my grassland but filling my ditches. He did use radish as a cover crop which worked a treat but never repeated it. |
| - We already do this/only plough for weed control. |
| - The problem with minimum tillage is you don't get such good yields. |
| - It's ok to spend some money as long as it works. |
| - Not willing. |
| - Getting the machines to do this is the key. |
| - There are occasions when we have to plough. We’ve tried not doing this before and faced crop failure. Also, where we apply manure, it needs to be incorporated. |

| **Table 11. Quotes and paraphrasing of farmers’ explanation for willingness to plant more trees and hedgerows on field.** |
| --- |
| - Very open to increasing biodiversity and we are forever gathering acorns, walnuts, pinecones etc |
| - Environmental protection |
| - I have small enclosures and well grown hedges, so a good tree covers already. Using BN5 CS I find my laid hedge over 15 years lasts as long as fencing without the depressing task of taking up old fencing. I use the wood for cooking and heating farmhouse and have surplus firewood. I can store the surplus dry and end up burning wood which has already replaced itself in the hedge. |
| - Not willing to because as trees grow taller, they block the sunlight from crops, and the fertility rate of crops around the trees is low, affecting the yield |
| - Not willing to. |
| - Hedgerows yes, trees no. Most corners that could have trees have already been planted. |
| - Have planted many trees and hedges - always wanting to do more. Benefits to the farm and the environment - a no-brainer. However, as we're a very small farm in today's terms, we wouldn't be able to do this on our productive land |

| **Table 12. Quotes and paraphrasing of farmers’ explanation for willingness to use some of your agricultural waste to produce bioelectricity?** |
| --- |
| - We don’t have any agricultural waste. Any off-spec produce goes to animal feed, thus producing good quality edible protein. Be delighted to investigate small scale on-farm incineration to produce power, however current govt policy appears to be to reduce self-reliance. That is where change is required. |
| - Need to sort the distribution and storage of electricity too. |
| - Too expensive to set up, cost is the hurdle. |
| - I would need to learn more about it |
| - Using waste straw to generate electricity is good for the country and the people. |
| - No idea how you do it and more importantly how expensive it is |
| - Never feasible for us - we don't have the scale |
| - Have a wind turbine and solar panels - am already a net electricity exporter |

| **Table 13. Quotes and paraphrasing of farmers’ explanation for willingness to plant bioenergy crops.** |
| --- |
| - do not think landlord would consent this. |
| - Feel nervous about bioenergy crops and the distortions experienced so far. Coming from the SW we have a very ancient and intimate landscape, so need to choose which crops with care. AD fed by maize, for example is fraught with environmental risks from beginning to end. |
| - I believe it’s a flawed idea |
| - I don't believe the figures, the fuel used to harvest and carting these crops, wear and tear on the roads and machinery, amount to more than any imagined savings. |
| - It quadruples the organic carbon in the soil, and it reduces pollution which I love, and it saves me money |
| - Local attempts have usually not been very successful. Partly due to contracts |
| - There must be a market, and a contract |
| - Unlikely - again because of our size. Wouldn't completely rule it out, though |
| - This is a shift away from growing crops |

| **Table 14. Quotes and paraphrasing of farmers’ explanation for willingness to would you be to change land use on your farm, e.g., converting arable land to extensive grassland?** |
| --- |
| - Not willing |
| - We use the method of plots in turn - fallow, let the land fully rest, so that it does not hurt the land |
| - Despite being very keen on maximising biodiversity and mitigating climate change I feel very uncomfortable about UK importing more and more food. By changing diets, we could sustain ourselves better, e.g. grow more vegetables and less sugar beet and feed livestock on food by products etc As a hobby farmer with rare breed cattle I feel that there is a lot of valuable cultural capital embodied in livestock farmers. I am also impressed at the variety and amount of good quality food/acre produced by experimental smallholders and the latest food research on plant diversity for the diet. Great potential there. |
| - The market will drive land use change. Diversity of crop is useful to combat adverse weather conditions e.g., in a drought wheat and barley can be taken earlier as a silage whole crop. This really helps to resolve drought issues |
| - We're already 100% pasture, so not applicable. Could convert to trees, though. |
